# Translation and trans-cultural adaptation to the Malay version of the COVID-19 vaccine hesitancy questionnaire among healthcare workers in Malaysia

**DOI:** 10.1371/journal.pone.0302237

**Published:** 2024-04-17

**Authors:** Siti Nur Aisyah Zaid, Azidah Abdul Kadir, Norhayati Mohd Noor, Basaruddin Ahmad, Muhamad Saiful Bahri Yusoff, Anis Safura Ramli, Jasy Liew Suet Yan

**Affiliations:** 1 School of Medical Sciences, Health Campus, Universiti Sains Malaysia, Kubang Kerian, Kelantan, Malaysia; 2 Hospital Universiti Sains Malaysia, Kubang Kerian, Kelantan, Malaysia; 3 School of Dental Sciences, Health Campus, Universiti Sains Malaysia, Kubang Kerian, Kelantan, Malaysia; 4 Faculty of Medicine, Universiti Teknologi MARA, Sungai Buloh, Selangor, Malaysia; 5 School of Computer Sciences, Universiti Sains Malaysia, Gelugor, Penang, Malaysia; Universiti Malaysia Sabah, MALAYSIA

## Abstract

**Introduction:**

Healthcare workers play a crucial role in supporting COVID-19 vaccination as they are the most trusted source of information to the public population. Assessing the healthcare workers’ hesitancy towards COVID-19 vaccination is pertinent, however, there are limited validated tools to measure their hesitancy on COVID-19 vaccines. This study aims to adapt and validate the first COVID-19 hesitancy scale among healthcare workers in Malaysia.

**Materials and methods:**

This study adapted and translated the Vaccine Hesitancy Scale (VHS) developed by the WHO SAGE Working Group. The scale underwent a sequential validation process, including back-back translation, content, face, and construct validity for Exploratory Factor Analysis (EFA) and Confirmatory Factor Analysis (CFA). The reliability was tested using internal consistency (Cronbach’s alpha composite reliability (CR) and average variance extracted (AVE)).

**Results:**

The data for EFA and CFA were completed by a separate sample of 125 and 300 HCWs, respectively. The EFA analysis of the C19-VHS-M scale was unidimensional with 10 items. A further CFA analysis revealed a uniform set of nine items with acceptable goodness fit indices (comparative fit index = 0.997, Tucker-Lewis index = 0.995, incremental fit index = 0.997, chi-squared/degree of freedom = 1.352, and root mean square error of approximation = 0.034). The Cronbach’s alpha, CR and AVE results were 0.953, 0.95 and 0.70, respectively.

**Conclusions:**

The questionnaire was valid and reliable for use in the Malay language.

## Introduction

The COVID-19 outbreak started early in the year 2020 and it has caused worldwide devastation through loss of life, well-being, and sustenance. As soon as it was declared a pandemic, pharmaceutical companies raced to develop vaccines against the virus leading to several versions being ready for distribution by the end of the year 2020. While the vaccination plan to end the pandemic was welcomed in most nations, some population segments resisted due to trust issues [1].

Vaccine hesitancy is described by the Strategic Advisory Group of Experts on Vaccine Hesitancy (SAGE) as an intentional delay or refusal of immunisation when vaccination services are readily accessible [2]. The World Health Organization (WHO) considered the phenomenon as one of the ten threats to global health in 2019 and recommended further research better to understand the topic [3–5]. Amidst the unclear reasons for hesitancy toward COVID-19 vaccines, the implications of vaccine hesitancy, such as reduced herd community and increased disease spread, are expected to be greater. The global prevalence of COVID-19 vaccination hesitancy is estimated at 25% (95%CI: 19%, 32%) and it is similar to that among healthcare workers (HCWs) (26%) and students (25%) [6]. Vaccine hesitancy among HCWs is worrying as they may provide inaccurate information and further discourage vaccination; hence, identifying and re-educating them is essential [6, 7]. The COVID-19 vaccines can further intensify disparities in vaccine acceptance [8], besides the fact that vaccine hesitancy is an old issue. Still, vaccine hesitancy has depolarized the vaccine supporters and their anti-vaccine counterparts.

The Vaccine Hesitancy Scale (VHS) is one of the instruments for assessing hesitancy towards vaccination and was developed by the WHO SAGE Working Group on Vaccine Hesitancy for use in various populations [9]. It was initially developed to assess parental hesitancy towards childhood vaccination using ten items in the English language. Respondents are asked to indicate a statement that closely describes their perceptions of each item using a 5-point Likert scale ranging from strongly disagree (1) to strongly agree (5). It is a versatile and reliable scale that can be adapted to various situations and has undergone validation and psychometric assessment [10, 11]. The instrument has been modified by Akel et al. as the adult Vaccine Hesitancy Scale (aVHS) for assessing the relationship between vaccine hesitancy and SAR-CoV2 in adults in the United States and China [12].

At present, there is no specific study on hesitancy towards COVID-19 vaccines among HCWs in Malaysia, plausibly related to the unavailability of validated instruments. Hence, this study aims to adapt and validate the Malay version of VHS specific for COVID-19 (C19-VHS- M) with specific applications for healthcare workers. The availability of the instrument can aid in identifying individuals and assessing the levels and concerns contributing to hesitancy that can be used in planning intervention strategies.

## Materials and methods

This study used aVHS in developing the COVID-19 Vaccine Hesitancy Scale Malay version (C19-VHS-M). The process of adapting and validating the scale was carried out in Phase I and Phase II, respectively. Permission to use the aVHS in this study was obtained from the author.

### Phase I: Adaptation and translation process

The adaption process was carried out by the research team and follows the guideline in a study by Sousa V.D. & Rojjanasrirat W. [13]. First, the cultural relevance of the reference instruments including the evaluation of suitability and meaning of the content, concepts, and terminology in the Malaysian context was assessed in a series of communications via Webex platforms and email exchanges. Following the agreement on the terms and phrases suitable for use in Malaysia, the translation into the Malay language for healthcare applications was then carried out by two independent certified translators familiar with Malaysian culture and linguistics; one was a specialist in medical sciences and the other, was in health sciences. The forward translations from English to the Malay language by the two translators were then examined by a third bilingual translator with experience in teaching the Malay language to identify ambiguities and inconsistencies in the use of vocabulary, sentence structure, grammar, and overall meaning in relation to the original instruments.

Harmonization of the translation was carried out by four research team members (2 specialists in family medicine, a statistician, and a nurse) who discussed and resolved all discrepancies and ambiguities in the translations through consensus decision-making, this resulted in the provisional version of C19-VHS-M. Next, two bilingual individuals who speak the English and Malay languages, a medical doctor familiar with the context and an English language teacher unfamiliar with the instrument but had no experience with the original VHS version were asked to translate the provisional version into the English language. Finally, the back-translated versions of the provisional C19-VHS-M were reviewed by the research team members who addressed and resolved issues relating to ambiguity and discrepancy in the cultural meaning and expression of the words and phrases of the items and response format before and the prefinal C19-VHS-M version was derived. The version has nine positively and one negatively worded item with the latter coded in reverse.

### Phase II: Validation process

#### Content and face validity

The validation process involved the assessment of content validity, face validity, construct validity using exploratory and confirmatory factor analysis, and reliability (internal consistency). The content validity of the prefinal version was assessed qualitatively and quantitatively on six HCWs including experts in family medicine (n = 3), paramedics (n = 1), and community medicine specialists (n = 2). Quantitative assessment was evaluated using the content validity index (CVI) by asking the experts to rate each item in the prefinal version based on a 4-point rating scale ranging from not relevant (1) to highly relevant (4) and calculating item-CVI (I-CVI) and scale-CVI (S-CVI). I-CVI and I-FVI at 0.79 or above suggest relevance to the domain, clarity, and comprehensibility for the intended target group [14]. Based on the recommendations of the experts, modifications to the items were made to the pre-final version and the process was repeated. The content validity was conducted from 11^th^ May 2022 until 21^st^ July 2022. Two rounds of content validity were conducted.

Then, the face validity study was conducted from the 1^st^ to the 14^th^. August 2022 to determine the clarity, comprehensibility, readability, and feasibility of the instrument among the HCWs. This study recruited HCWs from Hospital Universiti Sains Malaysia (HUSM) (n = 30) to evaluate the instrument using face-to-face interviews. They were instructed to provide additional explanations of their understanding and elaborate on the difficulties while assessing the items. For each item, they were asked to rate the clarity and comprehensibility based on a 5-point Likert scale ranging from not clear/comprehensible (1) to highly clear/comprehensible (5). The item-face validity index (I-FVI) was calculated by totalling the number of HCWs rated 4 or 5 and dividing it by the sample size. The S-FVI/Ave used the sum of the I-FVI divided by the sample size. Instruments with a Face Validity Index (FVI) greater than 0.79 are considered to have good clarity and comprehensibility [15]. Following, changes were made accordingly based on the comments of the respondents and a final version of the C19- VHS-M was prepared.

#### Construct validity and reliability

The construct validity of the final version was examined using data from a cross-sectional study on HCWs in Malaysia. This study included HCWs as defined in WHO 2022 and included doctors, dentists, nurses, health assistants, laboratory technicians, radiology technicians, dietitians, physiotherapists, health managers, and support workers including cleaners, drivers, and hospital administrators [16]. Also included were professional clinical postgraduate students who provide healthcare services during their studentship. The participants were Malaysian citizens in permanent or contract positions and able to read and comprehend Bahasa Malaysia.

Two separate samples were recruited for Exploratory Factor Analysis (EFA) (n = 125) and Confirmatory Factor Analysis (CFA) (n = 300). The sample size for EFA was calculated based on the subject-to-items ratio of 10:1 and accounting for 20% drop-out [17]. For the CFA, it was based on Comrey and Lee which indicates samples with n = 100, 200, 300, 500 and 1000 as poor, fair, good, very good, and excellent, respectively [18]. The data collection method was similar for both samples for EFA and CFA. The EFA data collection was conducted from 20^th^ August to 20^th^. August to 20^th^ Sept 2022 and the CFA data collection was performed from 20^th^ Oct to 20^th^ Nov 2022.

Samples were recruited using the chain-referral sampling method by distributing the the survey link invite to the social contact of researchers via multiplatform messaging apps such as WhatsApp and Telegram, or face-to-face methods. The recipient of the link was requested to further distribute the invitation to all their contacts throughout the country. The participants were informed that their participation was voluntary and provided their consent before starting the survey which lasted between 15 to 20 minutes. The test-retest reliability was not conducted in this study due to constraints in time and finance.

The reliability of a questionnaire may be conceptualized as the degree of consistency or stability exhibited by the survey findings. Measurement inaccuracy is a prevalent issue in content sampling, respondent changes, and variations across raters. To assess the dependability of a questionnaire, this study employed the internal consistency (Cronbach’s alpha coefficient), composite reliability (CR) and average variance extracted (AVE). AVE is the average amount of variation determined by the variables within a given construct or domain.

### Statistical analysis

The dataset can be retrieved from https://opendata.usm.my/handle/123456789/74704. The EFA was performed using the Principal Axis Factoring with Promax rotation and criterion set at Eigenvalue ≥ 1. The variables data were entered from Excel to the SPSS software. The sample adequacy was determined by the Kaiser-Meiyer-Olkin (KMO) value (>0.5) [19] and the significant level of Bartlett’s sphericity test at p<0.05 [19]. The analysis was carried out using the IBM SPSS Statistic version 26.0.

The CFA was performed using Analysis of Moment Structure (AMOS) software version 28.0. The purpose of the CFA is to assess the indicated domain in the EFA. In the context of the CFA methodology, it is common to construct route diagrams that depict the relationships between variables, as well as to estimate covariances and loadings of the observed or measured variables, which are often referred to as factors.

The CFA model that has been initialized is then executed and evaluated, with the exclusion of unimportant components that possess standardized regression weights less than 0.5 [20]. According to the suggestions, a good model fit may be determined by the following criteria: normal chi-square per degree of freedom (Cmin/df) < 3 (P value of > 0.05), Comparative fit index (CFI), Incremental fit index (IFI), and Tucker-Lewis index (TLI) ≥ 0.9 and Root-mean-square error approximation (RMSEA) ≤ 0.08 [18]. On the other hand, the modification indices (MI) were used to evaluate the goodness of fit. A high MI value suggests that there is a significant amount of redundancy present in a pair of variables.

The internal consistency of C19-VHS-M was evaluated by figuring out Cronbach’s α coefficient value which was measured using SPSS for the whole questionnaire and each of its subscales. A coefficient ≥0.7 indicated good internal consistency for the questionnaire [21]. Both CR and AVE were determined through CFA analysis and manually computed using established formulas found in published literature [22, 23]. The CR ≥ 0.6 and AVE >0.5 were acceptable to reflect satisfactory internal consistency [22].

### Ethical approval

The study was ethically approved by the Research and Ethical Committee, School of Medical Sciences, Universiti Sains Malaysia (USM/JEPeM/21100700) and Malaysia Ministry of Health (NMRR ID-21-02113-ZGG (IIR)). All participants provided informed consent. Written informed consent was used for the content validity and face validity study. Implied informed consent for EFA and CFA study. The procedure for the implied informed consent is that the information of the study and implied consent form was uploaded on the first page of the survey link via the Google form. The consent is in Bahasa Malaysia and it was stated clearly that this was implied consent. Subjects who click on ’I agree’ and complete the survey will be deemed to have consented to participate.

## Results

### Content and face validity

The I-CVI (score = 0.67 to 1) and S-CVI/Ave (0.92) scores of the first content validation process did not meet the cut-off level. Thus, the items were revised, and the content validity process was repeated; the result showed that the prefinal C19-VHS-M has an I-CVI ranging from 0.83 to 1 and S-CVI/Ave = 0.97 [14]. For the face validity, the analysis showed the I-FVI ranges from 0.93 to 1 and the S-FVI/Ave = 0.98. Both I-CVI and I-FVI were above 0.79, indicating that the questionnaire items are important, clear, and easy to understand for the HCW [15]. Several modifications also were made to a few items in response to the suggestions provided by the participants. The data for the final content and face validity were demonstrated in Table 1.

**Table 1:** Content and face validity for C19-VHS-M.

Coding	Items	I-CVI	I-FVI
A1	Vaksin COVID-19 ini penting untuk kesihatan saya. *The COVID-19 vaccine is important for my health*.	1	1
A2	Vaksin COVID-19 ini berkesan untuk mencegah jangkitan COVID-19 yang teruk. *The COVID-19 vaccine is effective in preventing serious COVID-19 infection*.	1	0.97
A3	Vaksin COVID-19 penting untuk kesihatan masyarakat bagi mencegah jangkitan COVID-19. *The COVID-19 vaccine is important for public health to prevent COVID-19 infection*.	1	0.97
A4	Semua vaksin COVID-19 yang disyorkan oleh Kementerian Kesihatan Malaysia (KKM) adalah bermanfaat. *All COVID-19 vaccines recommended by the Ministry of Health Malaysia (MOH) are beneficial*.	1	1
A5	Vaksin COVID-19 adalah selamat. *COVID-19 vaccines are safe*.	1	0.93
A6	Maklumat yang saya terima mengenai vaksin dari Kementerian Kesihatan Malaysia (KKM) boleh dipercayai. *The information which I received from the Malaysian Ministry of Health (MOH) is reliable*.	1	1
A7	Vaksin COVID-19 melindungi saya daripada jangkitan COVID-19. *This vaccine protects me from COVID-19 infection*.	0.83	1
A8	Secara amnya, saya bersetuju dengan perkara yang disyorkan oleh Kementerian Kesihatan Malaysia (KKM) tentang vaksin COVID-19. *In general*, *I agree with the recommendations made by the Malaysian Ministry of Health (MOH) regarding COVID 19 vaccine*.	1	0.97
A9	Saya bimbang terhadap kesan sampingan teruk vaksin COVID-19. *I am worried about the serious effects of COVID-19 vaccines*.	0.83	0.93
A10	Saya memerlukan vaksin COVID-19 sebagai langkah pencegahan menghadapi varian baharu COVID-19. *I need the COVID-19 vaccine as a preventative measure against the new variant of COVID-19*.	1	1

I-CVI–Item Content Validity Index

I-FVI–Item Face Validity Index

### Psychometric properties

The summary of the sample for the EFA (n = 125) and sample of CFA (n = 300) is presented in Table 2. In general, the mean (SD) age for EFA was 35.7(8.7) years while for CFA was 37.3 (8.4). The majority of the respondents were female, Malay ethnicity and Islam as the religion for both EFA and CFA may be due to the uniqueness of the population in Malaysia.

**Table 2:** Sociodemographic data of the participants.

Variables	EFA 125 n(%)	CFA 300 n(%)
Age (year)		
Mean (SD)	35.7 (8.7)	37.3 (8.4)
Gender		
• Male	25 (20.0)	70 (23.3)
• Female	100 (80.0)	230 (76.7)
Race		
• Malay	120 (96.0)	287 (95.7)
• Chinese	3 (2.4)	8 (2.7)
• Indian	2 (1.6)	3 (1.0)
• Siamese	-	2 (0.6)
Religion		
• Islam	120 (96.0)	287 (95.7)
• Buddha	3 (2.4)	10 (3.3)
• Hindu	2 (1.6)	3 (1.0)
Education		
• Primary School	-	2 (0.7)
• Secondary School	36 (28.8)	88 (29.3)
• Diploma/certificate	53 (42.4)	92 (30.7)
• Degree	33 (26.4)	110 (36.7)
• Master/PhD	3 (2.4)	8 (2.7)
Monthly Salary (RM)		
• <2500	40 (32.0)	75 (25.0)
• 2500–5000	44 (35.2)	127 (42.3)
• 5000–11000	39 (31.2)	87 (29.0)
• 11000–15000	-	7 (2.3)
• >15000	2 (1.6)	4 (1.3)
Marital Status		
• Married	97 (77.6)	238 (79.3)
• Single	28 (22.4)	62 (20.7)
Type of occupation		
• Safety Department	1 (0.8)	-
• Cleaning Department	6 (4.8)	1 (0.3)
• Pharmacy	2 (1.6)	28 (9.3)
• Forensic	1 (0.8)	-
• Nursing	53 (42.4)	102 (34.0)
• Public Health	1 (0.8)	13 (4.3)
• Nutrition and Dietetics	2 (1.6)	23 (7.7)
• Administration and Management	18 (14.4)	116 (38.7)
• Dentistry	2 (1.6)	-
• Medical	34 (27.2)	17 (5.7)
• Rehabilitation Medicine	5 (4.0)	-
• Medical Records	-	-
Duty State		
• Kelantan	103 (82.4)	268 (89.3)
• Terengganu	6 (4.8)	14 (4.7)
• Johor	4 (3.2)	2 (0.7)
• Pulau Pinang	3 (2.4)	3 (1.0)
• Kedah	2 (1.6)	2 (0.7)
• Pahang	2 (1.6)	3 (1.0)
• Selangor	2 (1.6)	3 (1.0)
• Perlis	1 (0.8)	1 (0.3)
• Negeri Sembilan	1 (0.8)	-
• Melaka	1 (0.8)	-
• Perak	-	3 (1.0)
• Sabah	-	1 (0.3)

### Construct validity and reliability

#### Exploratory Factor Analysis (EFA)

Of the 125 respondents, there was no missing data or missing item responses. Statistical significance of the Bartlett test of sphericity (<0.05) was sufficient for each item and the KMO measure of sampling adequacy was 0.918. The results suggest that the data met the necessary criteria for further factor analysis. Parallel analysis and scree plots all suggest two factors should be retained. Since the parallel analysis revealed a 2-factor model, thus EFA was carried out with two components. Therefore, a total of 10 items within the domain were subjected to EFA. Following rotation, the factor accounted for 66.5% of the total variance. The coefficient alpha was 0.91. However, only item A9 had a lower communalities value (<0.3).

Based on the eigenvalue, the number of constructs was set to one after item A9 was removed. The 1-factor model’s validity was further substantiated by the communalities of each characteristic since all factor loadings were above 0.3. After fixing to a factor, it accounted for 73.7% of the total variance and the factor’s internal consistency (Cronbach alpha) reliability was 0.95. Given these results, the 1-factor solution was accepted as the most adequate for representing the structure of C19-VHS-M with the participants. Table 3 shows the EFA descriptive statistics and communalities of C19-VHS-M.

**Table 3:** EFA for C19-VHS-M.

Items	Descriptive Statistics				Communalities
	Mean	SD	Skew	Kurtosis	
A1	4.16	0.72	-0.51	-0.65	0.79
A2	4.22	0.71	-0.35	-0.94	0.77
A3	4.19	0.76	-0.68	0.09	0.84
A4	4.05	0.82	-0.80	0.89	0.79
A5	3.94	0.81	-0.46	-0.17	0.78
A6	4.04	0.72	-0.45	0.14	0.80
A7	3.78	0.93	-0.72	0.30	0.52
A8	3.95	0.80	-0.68	0.83	0.80
A9	2.58	0.99	0.38	-0.07	0.09
A10	3.89	0.84	-0.72	0.67	0.69

#### Confirmatory Factor Analysis (CFA)

The confirmatory analysis demonstrated that the original 10 items of C19-VHS-M were not fit. This is due to item A9 having low factor loadings and had been removed. Then, six items were set as free parameter estimates, one pair at a time (A1-A2, A2-A3, A1-A3, A7-A8 and A7-A10). It was due to the high MI which is greater than 15. A final model that consists of 9 items signified the model fit (S1 Fig). The p-value for this analysis was 0.125 while the Chi-square/df was 1.352. Also, the CFI = 0.997, TLI = 0.995, IFI = 0.997, NFI = 0.989, GFI = 0.979 and RMSEA = 0.034 showed the good model fit. The goodness of fit indices provided evidence that the model exhibited a strong construct [20]. The standardized factor loadings were from 0.73 to 0.92 demonstrating that every item was significantly associated with the construct measurements. S1 Fig shows the final model for the questionnaire.

#### Reliability

The internal consistency of the questionnaire was established by the Cronbach’s α coefficient. The reliability coefficient was 0.953, which was a good internal consistency. The CR value was 0.95 and the AVE value was 0.70 for the construct, which showed a good measure of reliability.

## Discussion

A successful COVID-19 mass vaccination program relies heavily on people’s willingness to get vaccinated. Mass vaccination programme for COVID-19 infection depends on vaccine acceptance and vaccination acceptance is vital to ensure successful impact. Although the persistent presence of vaccination hesitancy is a public health concern, it continues to be shared among HCWs. HCWs worldwide play an essential part in the protection and treatment of patients and can influence patients’ decisions about vaccination. Understanding that HCWs in Asia, especially South Asia, are hesitant to get vaccinated is crucial because the rates of COVID-19 illness and death vary around the world [24]. They have been regarded as a priority group for COVID-19 vaccination [25], just as in Malaysia. The mentioned matter gained attention because the government implemented mandatory COVID-19 vaccination measures [26]. The mandatory COVID-19 vaccination in Malaysia is only for government employees (including HCWs) with exceptions for those with health issues. This study addresses the gap in understanding the HCWs’ attitudes and perceptions of the COVID-19 vaccine using a newly adapted and translated questionnaire.

The present study aims to evaluate the psychometric features of the Malay version of the C19-VHS, a modified form of the VHS. The primary focus of this assessment was to measure hesitation, primarily related to COVID-19 vaccinations. Using a rigorous and structured approach, we have successfully devised a questionnaire through validation procedures, ensuring its reliability and accuracy. Overall, the questionnaire was suitable to use for the Malaysian culture. This tool is designed to be brief and easy to use, making it simple for respondents to understand.

The initial C19-VHS-M consists of 10 items but remain 9 items after the validation process. Validating the questionnaire ensures it measures what it’s supposed to and provides excellent evidence. Numerous studies have been conducted [4, 27] to evaluate the vaccination reluctance among HCWs. However, there is a paucity of evidence on the questionnaire’s validity and reliability, particularly in terms of its cultural and linguistic appropriateness for the target group. The C19-VHS-M was translated, adapted, and validated in the local language to make it applicable and usable for all categories of HCWs in Malaysia. A very recent study in Israel also reported the psychometric properties of the C19-VHS, but the study was conducted in early 2021 and done among the public population [28]. It was different with this study since it was done at the end of 2022 among HCWs.

We employed both qualitative and quantitative methods for content and face validity in the early validation of the C19-VHS-M questionnaire. Similarly, a study from Singapore [29] also did both content and face validity by five domain experts and targeted respondents respectively. We evaluated both options and decided to include only questions with an I-CVI score of 0.79 or above in the questionnaire. In addition, we also utilised enough experts for the content validation and this approach supports the evidence that the items were essential and relevant for measuring the reluctance of HCWs to receive the COVID-19 vaccine.

A face validity assessment was implemented among HCWs to evaluate the clarity of the questions from the perspective of the intended users. The experts in the field may hold different opinions about the relevance and comprehension of the items. The majority of items obtained scores over 0.90 on the I-FVI scale, and the S-FVI/Ave for this questionnaire is 0.98, suggesting that all the items were perceived as clear and comprehensible. The alteration was also made to enhance clarity, taking into account the feedback provided by the respondents. In general, the C19-VHS-M questionnaire experienced improvements through the content validity and face validity procedures, resulting in significant modifications to the questions, ideas, language, and overall structure of the instrument.

Ultimately, the C19-VHS-M demonstrated good psychometric properties. The EFA analysis indicated one factor which explained a total variance of 73.69%, higher than the original VHS [11]. Consequently, the questionnaire becomes one-dimensional. It differed from the other validation studies that produced two factors due to different target populations [28]. After running the analysis, 1 item over ten items was removed due to factor loading less than 0.3. Thus, only 9 items proceed to the CFA process.

The CFA technique is employed to assess and validate the factor structure of a measuring instrument. CFA, essentially a model testing technique, starts the analytical process by formulating a hypothesis grounded in vigorous theoretical and empirical foundations [30]. In contrast, the application of EFA is employed to investigate the potential latent factor structure of a given measuring instrument. The committee in this study decided to keep the original 1 factor with 9 items similar to the result produced from the EFA, 1 domain with 9 items. There were 6 items set as free parameter estimates due to high MI. The panel evaluated them and decided not to remove the items because they may contain significant or meaningful content. In this study, reliability analysis indicated that the internal consistency was adequate, providing a better Cronbach’s alpha (0.953) than in a recent study [11, 28]. Hence, it shows that the questionnaire is valid and reliable to assess HCW hesitancy on COVID-19 vaccines. In addition, both CR and AVE for this questionnaire were acceptable showing a good level of internal consistency.

We believe this study contributes to the new evidence of validated methods for assessing healthcare workers’ reluctance towards COVID-19 vaccines. The questionnaire items in this study are relevant to the issue of vaccine hesitancy among healthcare professionals. Besides, a key focus of this research is ensuring that the anonymity and privacy of the participants are carefully safeguarded. The researchers understand that HCWs face unique vulnerabilities and risks due to their profession, unlike other respondents like the general population. Hence, the research was conducted discreetly, outside official channels, given the sensitive nature of the topic. One of the main strengths of this study was that HCWs could participate voluntarily, without any pressure. Next, to ensure the accuracy of our data collection, we employed various methods and resources, starting with EFA and then moving on to CFA. Additionally, we restricted access to the Google Form to allow participants to fill it out only once. However, despite our efforts, we couldn’t entirely control the results, as there might be overlapping responses that could impact the findings.

There are a few limitations of this study. The adaptation of the instrument limits its utilisation to a population who understands the Bahasa Malaysia language. The sampling method is not random, has fewer participants from the lowest and the highest education levels, and more participation from a certain state, hence may not be representative of the Malaysian population; nevertheless, the instrument uses the standard language that can be understood across the socioeconomic status, and ethnic and cultural background in the country. The sampling method is an acceptable data collection process and has been used in earlier studies [31, 32]; it has the advantage of reaching a wider sampling frame, rapid distribution, and lowering the cost of study but limited by the response rate. The test and re-test reliability was not conducted because the data collection did not record the personal information for re-administering the questionnaire; despite the lack of evidence for consistency over time, the instrument showed good internal consistency. Despite the limitations, findings suggest that the C19-VHS-M possesses potential use in assessing COVID-19 hesitation among healthcare workers in Malaysia. This assertion is supported by the favourable outcomes seen during the questionnaire’s adaptation, translation, and validation processes.

## Conclusion

This study demonstrated that the C19-VHS-M is a valid and reliable self-administered instrument for assessing vaccine hesitancy in healthcare workers in Malaysia.

## Supporting information

S1 Fig CFA for C19-VHS-M. (TIF)
