# Supplementary figures and images for: Translation and trans-cultural adaptation to the Malay version of the COVID-19 vaccine hesitancy questionnaire among healthcare workers in Malaysia
Source: PLoS One. 2024 Apr 17;19(4):e0302237. doi: 10.1371/journal.pone.0302237 (PMC11023593; doi:10.1371/journal.pone.0302237)

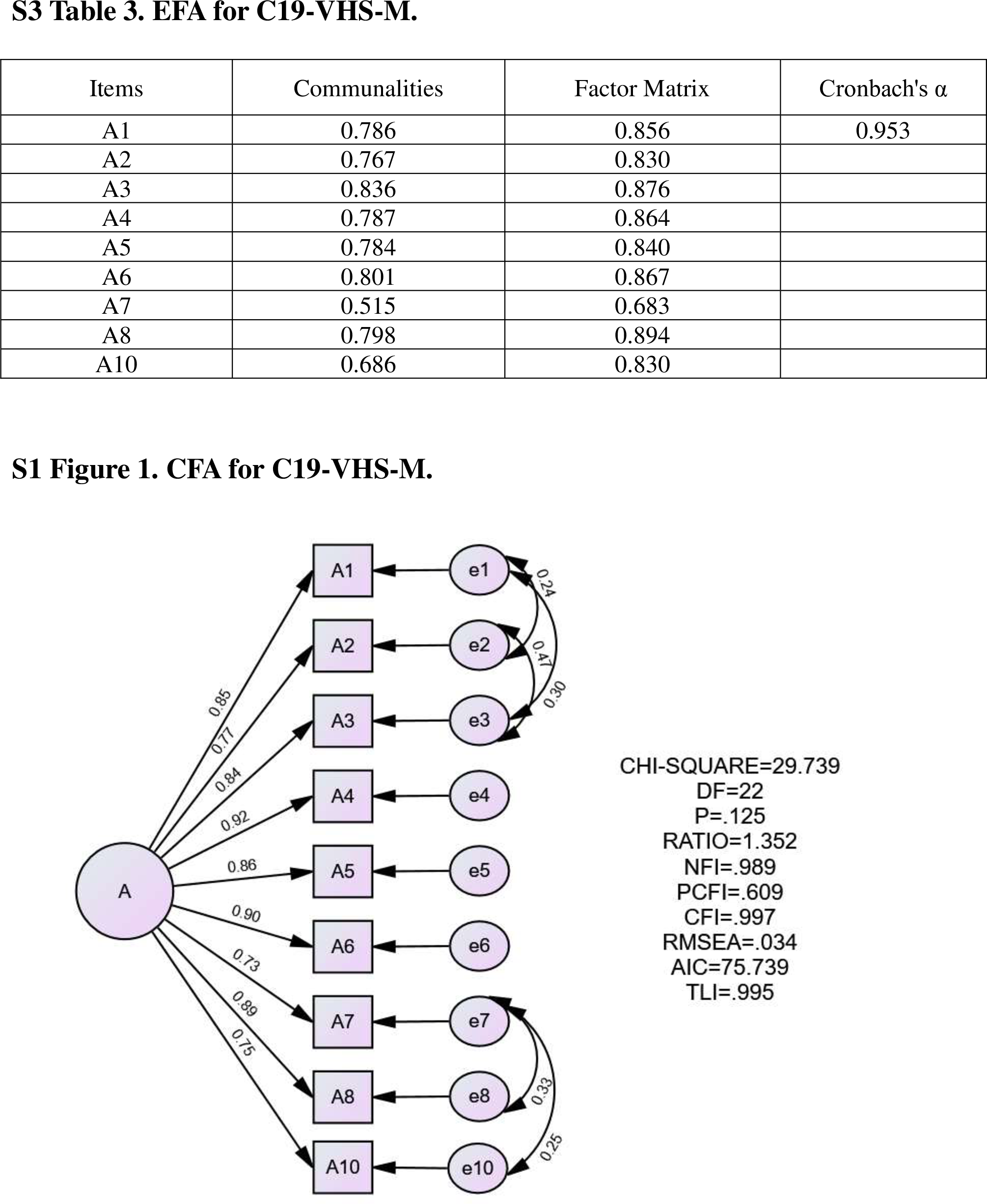

Supplement: S1 Fig — (TIF) [file pone.0302237.s001.tif]
